# Supplementary material for: Filming enhanced ionization in an ultrafast triatomic slingshot
Source: Commun Chem. 2023 Apr 27;6:81. doi: 10.1038/s42004-023-00882-w (PMC10140156; doi:10.1038/s42004-023-00882-w)
Supplement: Supplementary file 2 — Supplemental Information (PDF) [file 42004_2023_882_MOESM2_ESM.pdf]

Supplementary Information for
Filming Enhanced Ionization in an Ultrafast Triatomic Slingshot

A.J. Howard *et al.*

Contents

SUPPLEMENTARY METHODS **1**

1 Experimental Methods **1**
1.1 The Jacobian Factor 1

2 Theoretical Methods **1**
2.1 Comparing Experimental and Theoretical Trajectories 1
2.2 Analysis of the 3-Dimensional Enhancement Volume 3
2.3 Comparing Single-Pulse and Double-Pulse KER Distributions 3

SUPPLEMENTARY METHODS

1 Experimental Methods

1.1 The Jacobian Factor

Equations 1a–1c break down when β = 180°. Here there is nothing to distinguish the x_m axis from the z_m axis since the momenta of the deuterons are exactly back-to-back. This is not a problem experimentally, however, because the probability of detecting two deuterons with exactly opposite momentum vectors is vanishingly small. This fact can be most easily visualized by imagining a 3-dimensional space (in x, y, and z) where the normalized momentum vector of one deuteron always defines the -z axis. The normalized momentum vector of the other deuteron could therefore exist anywhere on the surface of a 3-dimensional unit sphere. Finding the total distribution in β requires integrating over the surface area of this sphere. To do so, it is useful to use spherical coordinates where the differential surface area of the unit sphere (dA) can be written as dA = $2\pi\cos(\phi)$ dϕ, where ϕ is the polar angle to the z-axis. Here, $\cos(\phi)$ is called a "Jacobian factor" because it results from a change of basis to spherical coordinates. In this coordinate system, $\phi = \beta - 90°$, and so when $\beta = 180°$, $\phi = 90°$ and the Jacobian factor is zero. This factor explains the lack of counts at $\beta = 180°$. Importantly, the Jacobian factor has been corrected for (divided out) in all of the 1-dimensional angular distributions presented within the main text. This is why, for example, the 1-dimensional distributions of β and θ shown in Figs. 2g and 2h, do not tend toward zero at $\beta = 180°$ or $\theta = 0/180°$. However, this factor is still present in all other representations of the data, such as the 2-dimensional Newton plots shown in Figs. 2a–2e, leading to a noticeable lack of counts at $\beta = 180°$.

2 Theoretical Methods

2.1 Comparing Experimental and Theoretical Trajectories

To achieve the best possible agreement between theoretical and experimental observables, as displayed in Figs. 3a–3f, the theoretical populations on each of the nine states of D_2O^{2+} were optimized to best reproduce the data. This was accomplished by binning the theoretical trajectories coarsely to match the resolution of the experimental dataset, as seen in Fig. S1, then minimizing the difference between each time-resolved observable as the theoretical populations were varied. Specifically, we minimized the difference between Figs. S1a and S1d, Figs. S1b and S1e, and Figs. S1c and S1f simultaneously. The results are shown in Fig. S1g–S1i which display the 2-dimensional difference plots between theory and experiment following optimization. The accompanying populations on each state yielded by this analysis are shown in Table S1.

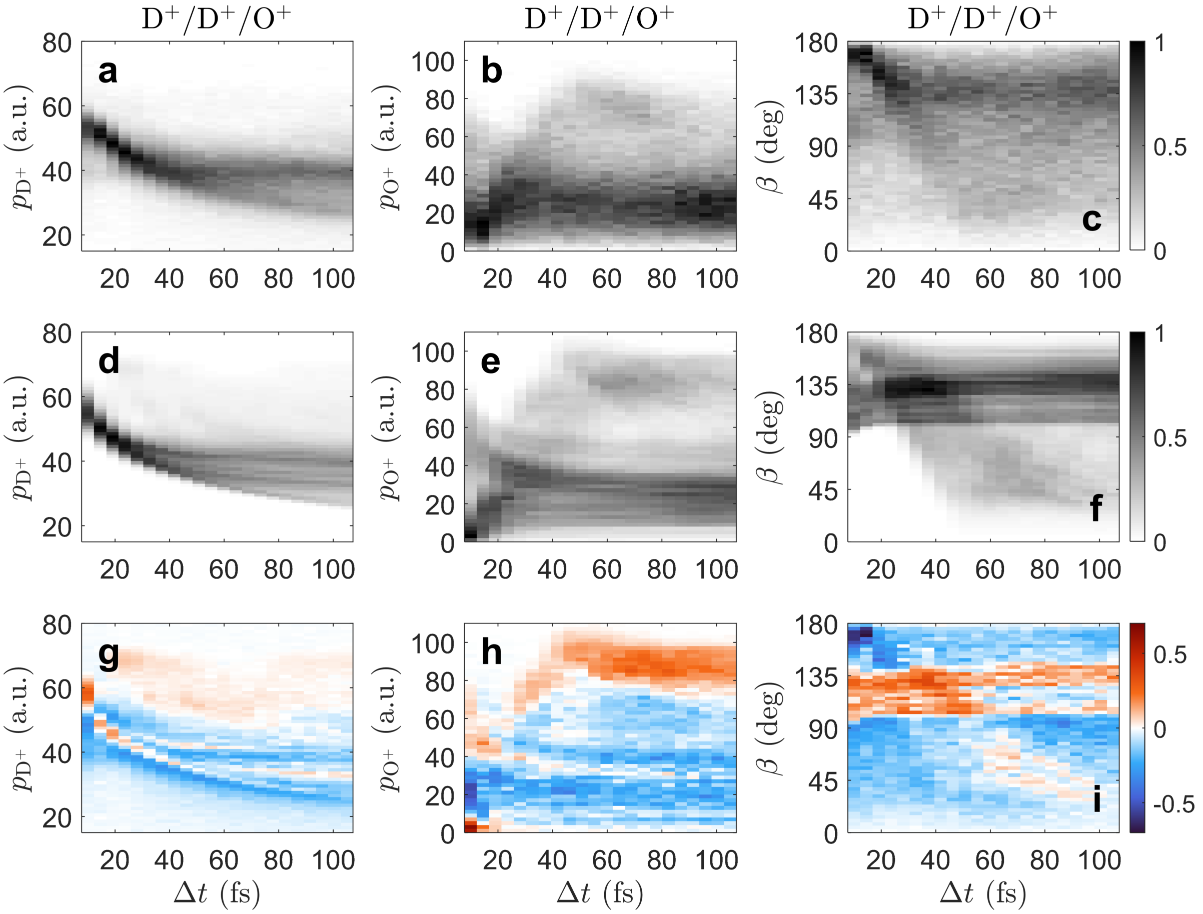

Figure S1: Comparing theoretical D_2O^{2+} dynamics to the data. (**a–c**) Two-dimensional histograms of the three experimental observables: the magnitude of the deuteron momentum (p_{D^+}), the magnitude of the oxygen-ion momentum (p_{O^+}), and the momentum-frame bend angle (β), each plotted as a function of interpulse delay Δt. (**d–f**) Two-dimensional histograms of the same three observables as in (a–c) but for a simulated ensemble of trajectories. The normalized populations of each state are listed in Table S1. (**g–i**) The difference between theory and experiment (difference = theory - experiment) for each of the observables seen in (a–c).

Figs. S1g–1i show that there is poor quantitative agreement between the data (Figs. S1a–S1c) and simulations (Figs. S1d–S1f), revealing regions in excess of 50% error. This is expected: the EI phenomenon is highly sensitive to the geometry as well as the occupied state of the molecule. The angular distribution in Fig. S1c, for example, has the most dense clustering of counts at $\Delta t \sim 18$ fs and $\beta \sim 150°$ (corresponding to $\theta_{\mathrm{DOD}} \sim 180°$). The Wigner distribution of trajectories launched on any combination of the nine dication surfaces could never reproduce this clustering, because EI acts as a strong selective filter. The closest match comes from populating the $2^1\mathrm{A}_1$ state, in which (according to Table 1) 74% of the trajectories undergo the rapid "slingshot" motion that is favored by EI. The frequent occurrence of slingshot motion on the $2^1\mathrm{A}_1$ state explains why this analysis, as seen in Table S1, yields a maximal population in this state; however, the other states are still necessary to reproduce all the features seen in the experiment. For example, populating the higher lying states (such as $^1\mathrm{B}_2$) is necessary in order to reproduce the motion in which β unbends from 100° to 140° as Δt progresses from 10 to 30 fs. Ultimately, the lack of quantitative agreement between theory and experiment is a manifestation of the EI phenomenon. Furthermore, the subset of states that are promoted to the trication can vary as a function of delay; the state population distribution extracted for early delays yields different results than those extracted for late delays, further complicating any quantitative analysis of the state populations. For these reasons, the populations displayed in Table S1 should not be considered an accurate depiction of the initial ensemble of dicationic states launched by the initial pulse in the pair.

State	Population
$^3B_1$	0.37
$1\,^1A_1$	0.49
$^1B_1$	0.02
$^3A_2$	0.11
$^1A_2$	0.13
$2\,^1A_1$	1.00
$^3B_2$	0.04
$^1B_2$	0.44
$3\,^1A_1$	0.25

Table S1: Theoretical populations of D_2O^{2+} extracted from the data. The normalized population within each dication state (labeled by C_{2v} symmetry) following optimization between experiment and theory. Optimization was performed to minimize the difference of the three following time-resolved observables: the magnitude of the deuteron momentum (p_{D^+}), the magnitude of the oxygen-ion momentum (p_{O^+}), and the momentum-frame bend angle (β). Here, the highest population is in the 2^1A_1 state.

2.2 Analysis of the 3-Dimensional Enhancement Volume

Before constructing a 3-dimensional space to localize the enhancement, we first applied a strict filter in time. To do so, we plotted the normalized yield of all $D^+/D^+/O^+$ coincidences as function of Δt and fit this distribution to a simple Gaussian. As seen in Fig. S2, this normalized yield is well approximated by a Gaussian distribution at early interpulse delays. We then defined our filter as the 6-fs window centered around the peak of this fit: (12 fs $< \Delta t <$ 24 fs). After applying this filter to all $D^+/D^+/O^+$ coincidences, we constructed a 3-dimensional histogram in Δt, β, and p_{D^+}. Drawing an isointensity surface at 50% of the maximum value in this 3-dimensional histogram yields the 3-dimensional enhancement volume pictured in Fig. 4a.

As stated in the main text, we then utilized this 3-dimensional enhancement volume in order to recover the geometry associated with EI. To do so, we propagated all 18,432 trajectories (2048 trajectories per state $\times$ 9 states) through this 3-dimensional space and assigned a weight to each trajectory per time-step (in Δt) based on the local value of the enhancement at that point within the 50% enhancement volume. The weight assigned outside of the enhancement volume was zero. If, for example, a trajectory passes through the global maximum of the 3-dimensional histogram, it is assigned a weight of 1 at that particular time-step.

The ultimate result of this analysis is a list of 10,212 trajectories (8220 were eliminated entirely), each of which carries a weight that is a function of Δt. The weighted sum of these trajectories was then used to generate the 2-dimensional histogram in r_{OD} and θ_{DOD} that is shown in Fig. 4b. As there is nothing experimentally distinguishing $r^{(1)}_{OD}$ and $r^{(2)}_{OD}$, Fig. 4b is the average between the 2-dimensional histograms of [$r^{(1)}_{OD}$ and θ_{DOD}] and [$r^{(2)}_{OD}$ and θ_{DOD}].

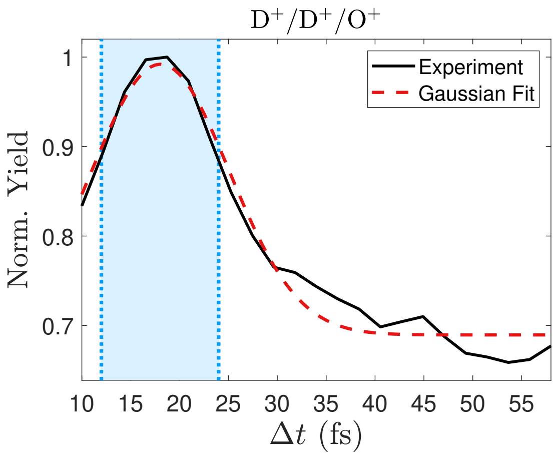

Figure S2: Delay-dependent yield of D_2O^{3+}. The normalized yield of $D^+/D^+/O^+$ coincidences as a function of interpulse delay Δt (in solid black) and a simple Gaussian fit to this yield (in dashed red). Highlighted in cyan is a 6-fs wide time-window centered around the interpulse delay at which the peak of the Gaussian fit occurs: Δt = 18 fs.

2.3 Comparing Single-Pulse and Double-Pulse KER Distributions

To address one possible reason for the disparity in KER between the three-body dissociations following formation of D_2O^{3+} via single pulses (where $\tau \geq$ 19 fs) and pulse pairs (where Δt = 18 fs), we make use of a simple model of CEI. In Fig. S3, we used the three-charge Coulomb repulsion potential (Eq. 3) to calculate the KER for the Coulomb explosion of a static and linear D_2O^{3+} molecule as a function of symmetric stretch of the OD bond length r_{OD}. Here we assume that the DOD bend angle is 180° because this is a prerequisite for the EI phenomenon described in the main text. According to this model, the peak of the

KER distribution measured using 19-fs single pulses (17.5 eV) equates to an OD bond length of 1.66 Å. However, using the same model to find the equivalent OD bond length for the peak of the KER distribution measured using pulse pairs with 18-fs delay yields 2.03 Å (see Fig. S3). This differs significantly from the critical OD bond length (2.2 Å) recovered in the main text. The reason for this disagreement is the additional kinetic energy accumulated by propagation on the states of D_2O^{2+}. By contrast, the simulated KER for the slingshot trajectory (the same trajectory shown in Figs. 2g–2k) takes account of this effect and correctly yields a KER of 17.5 eV for an OD bond length of 2.2 Å (see Fig. S3). This analysis therefore suggests that the KER found when ionizing with single pulses ($\tau \geq 19$ fs) may have a significant contribution (on the order of 1-2 eV) from the kinetic energy accumulated in the intermediate charge states (D_2O^+ or D_2O^{2+}). As a result, the OD bond length for this case is likely greater than 1.66 Å and may be closer to 1.8 Å.

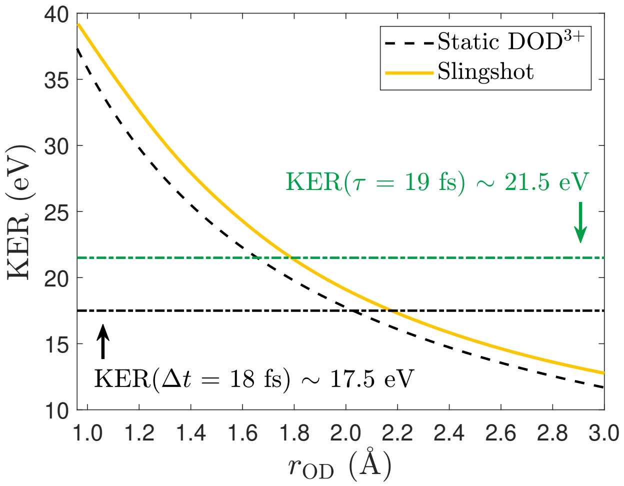

Figure S3: KER of D_2O^{3+} with and without intermediate dynamics. The total kinetic energy release (KER) as a function symmetric stretch of the OD bond (r_{OD}) for the three-body Coulomb explosion of a static linear DOD molecule (dashed black line) and the slingshot trajectory first seen in Figs. 3g–3k (solid yellow line). Two values of KER are represented as horizontal lines: 17.5 eV and 21.5 eV. These values correspond to the peak of the KER distribution for double pulses at a delay of Δt = 18 fs (black dash-dotted line) and single pulses with a duration of τ = 19 fs (green dash-dotted line) respectively.
